# Detection of bacterial sulfatase activity through liquid- and solid-phase colony-based assays

**DOI:** 10.1186/s13568-017-0449-3

**Published:** 2017-07-11

**Authors:** Hey Young Yoon, Hyung Jun Kim, Soojin Jang, Jong-In Hong

**Affiliations:** 1 Department of Chemistry, College of Natural Sciences, Seoul National University, Seoul, 08826 Republic of Korea; 2 Antibacterial Resistance Research Laboratory, Department of Discovery Biology, Institute Pasteur Korea, 16 Daewangpangyo-ro 712 beon-gi, Bundang-gu, Seongnam, Gyeonggi-do 13488 Republic of Korea

**Keywords:** Bacterial sulfatase, Activity-based probe, *N*-methyl isoindole, Colony-based assay, Liquid-phase assay, Solid-phase assay

## Abstract

Bacterial arylsulfatases are crucial to biosynthesis in many microorganisms, as bacteria often utilize aryl sulfates as a source of sulfur. The bacterial sulfatases are associated with pathogenesis and are applied in many areas such as industry and agriculture. We developed an activity-based probe **1** for detection of bacterial sulfatase activity through liquid- and solid-phase colony-based assays. Probe **1** is hydrolyzed by sulfatase to generate fluorescent *N*-methyl isoindole, which is polymerized to form colored precipitates. These fluorescent and colorimetric properties of probe **1** induced upon treatment of sulfatases were successfully utilized for liquid-phase sulfatase activity assays for colonies and lysates of *Klebsiella aerogenes, Mycobacterium avium* and *Mycobacterium smegmatis*. In addition, probe **1** allowed solid-phase colony-based assays of *K. aerogenes* through the formation of insoluble colored precipitates, thus enabling accurate staining of target colonies under heterogeneous conditions.

**Electronic supplementary material:**

The online version of this article (doi:10.1186/s13568-017-0449-3) contains supplementary material, which is available to authorized users.

## Introduction

Sulfur is a chemical element essential to all organisms, as it is required for the biosynthesis of cysteine and methionine; it is also involved in many redox reactions that take place in biological systems (Kertesz 2000). Microorganisms are capable of acquiring sulfur for biosynthesis by assimilating inorganic sulfates or organosulfur compounds, such as sulfonates and sulfate esters (Kertesz 2000; Stipanuk 1986). Bacterial arylsulfatases catalyze the hydrolysis of aromatic sulfate esters and participate in the metabolic pathways through which sulfur is procured by organosulfur compounds. Considering the ability to hydrolyze organosulfur compounds, bacterial arylsulfatases would be useful for many areas such as industry and agriculture (Stressler et al. 2016). In practice, bacterial arylsulfatases are applied in the desulfation of agar (Kim et al. 2004; Lim et al. 2004; Wang et al. 2015). Their activities are strongly influenced by bacterial growth environmental conditions and thus their measurements can be used for soil quality assessment (Garcia-Sanchez et al. 2016; Klose et al. 1999). In addition, it was suggested that sulfatase activity is related with the degradation of endosulfan, an extensively used insecticide (Kalyani et al. 2009; Narkhede et al. 2015). Recently, it was reported that sulfatases are potentially implicated in bacterial pathogenesis (Hickey et al. 2015). Furthermore, bacterial sulfatases might be involved in decomposition of sulfated mucins (Murty et al. 1992) and reconstruction of extracellular structures by desulfation of glycosaminoglycans (Mougous et al. 2002) for bacterial infection. However, despite of the various usages and the importance of sulfatase activity, only a few bacterial sulfatases were characterized.

Sulfatases contain the conserved Cys/Ser-X-Pro-X-Arg motif in their active sites. The first residue of the motif, which can be either cysteine or serine, is post-translationally modified to form C_α_-formylglycine (FGly), a unique amino acid that is the key catalytic residue for sulfate ester cleavage (Hanson et al. 2004; Knaust et al. 1998). In eukaryotes, the first residue of the motif is cysteine; inability to post-translationally modify this residue to form FGly in humans results in a rare lysosomal storage disease called multiple sulfatase deficiency (MSD) (Dierks et al. 2003; Diez-Roux and Ballabio 2005; Hanson et al. 2004). In prokaryotes, the first residue of the active site motif, i.e., the FGly progenitor, can be either cysteine or serine (Dierks et al. 1998; Marquordt et al. 2003; Miech et al. 1998). Sulfatases are believed to have either different FGly formation pathways or a common pathway with different modulating cofactors causing different localizations (Kertesz 2000). These reports indicate that post-translational modifications of sulfatases can regulate their activity, localization and/or stability, and all expressed sulfatases may not be capable of hydrolyzing sulfate esters (Hanson et al. 2004; Soufi et al. 2015). Thus, it is necessary to develop diverse assay methods for detecting sulfatase activity.

Determination of microorganisms expressing arylsulfatase requires a simple and easy assay method. Many methods for detection of arylsulfatase activity used bacteria cell lysates which were prepared through time–consuming and complicated processes. Colony-based assays are simple and thus can reduce sample preparation time. In addition, they also allow functional information to be acquired in an organism’s physiological environment (An and Tolliday 2010). Solid-phase assays are proper ways to screen and isolate the potential bacterial strains that express arylsulfatases. Solid-phase assays that exhibit sharp and clear image changes according to sulfatase activity are particularly useful in directly detecting individual colonies of interest (Baud et al. 2015; Green et al. 2014; Weiss et al. 2014) and they could offer easy methods for industrial applications (Bric et al. 1991; Kasana et al. 2008). Therefore, a colony-based solid-phase assay method would be most appropriate one to study bacterial arylsulfatases.

Activity-based probes, based on detecting specific enzymatic activity in a cellular context, are powerful tools for enzyme activity assays (Heal et al. 2011). Previously reported activity-based probes for sulfatase activity assays contained luminophores and sulfate esters, with sulfatase activity inducing an optical response (Beatty et al. 2013; Park et al. 2012; Rush et al. 2010; Smith et al. 2014). Although those probes were characterized by fast response times and low detection limits, they were deployed in purified enzyme solutions or bacterial lysates.

**Scheme 1:**
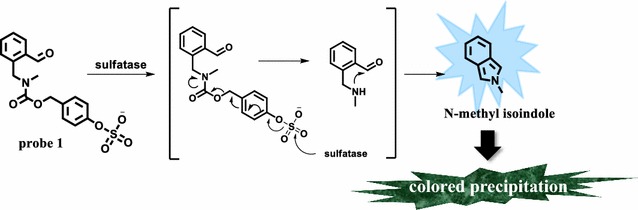
Strategy of probe **1** for sulfatase activity detection

We previously reported an activity-based probe **1** (Scheme 1), which enables detection of sulfatase activity in purified enzyme solutions through fluorescence enhancement (Yoon and Hong 2017). Probe **1** consists of sulfate ester as a substrate and benzaldehyde as a responsive unit, which are linked with a self-immolative moiety. The cleavage of the sulfate ester in probe **1** by sulfatase is followed by intramolecular cyclization, resulting in the formation of *N*-methyl isoindole which emits fluorescence at 415 nm. However, *N*-methyl isoindole is unstable and easily undergoes autooxidation and polymerization (Bonnett et al. 1973; Kochi and Singleton 1968; Rettig and Wirz 1976). The polymerization of *N*-methyl isoindole would trigger the formation of colored precipitates when a higher concentration of probe **1** was incubated with sulfatase for a longer period of time. These properties of probe **1** enabled us to detect sulfatase activity through liquid- and solid-phase colony-based assays (Scheme 1).

## Materials and methods

### Cultures and growth conditions

*Klebsiella aerogenes* was obtained from Hideko Urushihara at University of Tsukuba. *Mycobacterium avium* 104, and *Mycobacterium smegmatis* mc2-155 (ATCC 700084™) were obtained from Yonsei University. *Staphylococcus aureus* (ATCC 700698™) was purchased from American Type Culture Collection (ATCC). For the growth of *K. aerogenes* and *S. aureus*, Nutrient broth (NB) (BD Difco) was prepared according to the manufacturer’s instruction, and the media was supplemented with 1.5% (w/v) agar to make growth plates. For the growth of *M. avium* and *M. smegmatis*, 7H9 (BD Difco) media was prepared according to the manufacturer’s instruction and supplemented with 0.5% (w/v) BSA, 0.08% (w/v) NaCl, 0.2% (w/v) dextrose, 0.1% (v/v) tween 80, and 2% (v/v) glycerol to make the complete media. Also, 7H11 (BD Difco) growth plate was prepared according to the manufacturer’s instruction, and supplemented with 0.5% (v/v) glycerol, 0.05% (v/v) tween 80, 10% (v/v) OADC solution. The bacteria were grown at 37 °C, and the growth was monitored by the optical density at 600 nm.

### Enzymatic assays of purified sulfatase with probe **1**

Probe **1** was synthesized according to modified literature procedures (Yoon and Hong 2017).

Biochemical activity assays with *Helix Pomatia* sulfatase (Sigma, S9226) and *Aerobacter aerogenes* sulfatase (Sigma, S1629) were carried out in 96-well plates with the total volume of each plate being 250 μl at 37 °C and pH 7.4. 1 mM probe **1** and various amounts of sulfatase in 50 mM Tris buffer were used.

The detailed procedures were described in the supplementary information.

### Sulfatase activity assays of microorganisms with probe **1** in liquid-phase

For fluorescence reading, 250 µl of bacteria and isolated bacterial lysates in Tris buffer (50 mM, pH 7.4) were prepared in 96-well black microplates (Greiner). 2.5 µl of probe **1** (1 mM) was added to test wells, and the same volume of DMSO (1 mM) was added to control wells. The fluorescence intensity (λ_ex_ = 327 nm, λ_em_ = 415 nm) was measured at 37 °C in a time-dependent manner by the Victor3 multilabel plate reader (Perkin-Elmer) (0-240 min). For precipitation observations, 1 mM probe **1** with bacteria or bacterial lysates in Tris buffer (50 mM pH 7.4) were prepared in the 96-well white plate (Falcon) and incubated at 37 °C for 24 h.

### Solid-phase colony-based assays

We carried out the solid-phase assay with probe **1** following the published procedures (Baud et al. 2015; Green et al. 2014; Weiss et al. 2014).

Bacterial cultures were diluted to an OD_600_ of 0.1. Cellulose acetate membranes (Advantec MFS Inc. 0.2 μm pore size) were placed on the surface of an agar plate containing a growth medium. Then, the diluted cultures were plated onto the membranes, and the plates were incubated at 37 °C for 24 h (for 48 h for *M. avium*). Filter papers (Whatman) were soaked in a solution of probe **1** (5 mM) in 50 mM Tris buffer. The membranes were placed on top of the filter paper soaked in probe **1** solution, and incubated 24 h at 37 °C.

## Results

### Sulfatase activity tests

**Fig. 1:**
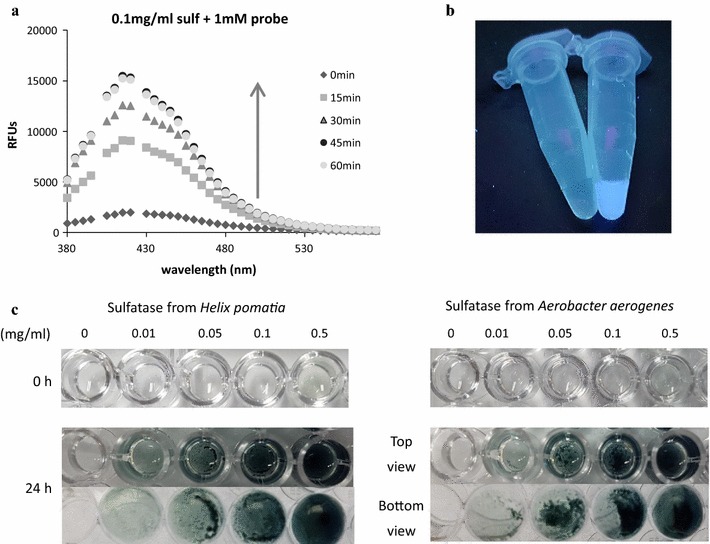
**a** Fluorescence intensity enhancement of probe **1** upon treatment with sulfatase from *Helix pomatia*, **b** fluorescence emission of probe **1** under UV lamp incubated with sulfatase from *Helix pomatia* for 30 min and **c** generation of colored precipitates after 24-h incubation; 1 mM probe **1** with sulfatase from *Helix pomatia* (*left*) and from *Aerobacter aerogenes* (*right*)

To evaluate the ability of probe **1** to detect sulfatase activity, we compared the fluorescence intensities and color changes of probe **1** in the presence or absence of commercially available arylsulfatases from *Helix pomatia* and *A. aerogenes*. We used 1 mM probe **1** and 0.1 mg/ml of sulfatase from *H. pomatia*, dissolved in 50 mM Tris buffer at pH 7.4. For about one hour after the initiation of the reaction with the sulfatase, the fluorescence intensity increased in a time-dependent manner (*Ф*_F_ = 0.146; the quantum yield was determined using tryptophan (*Ф*_F_ = 0.12 in water) as a standard) (Brouwer 2011). Then, the intensity decreased and colored precipitates formed spontaneously (Fig. 1; Additional file 1: Figures S1, S2).

### Calculation of kinetic parameters and detection limits of probe **1**

For the calculation of the kinetic values, we used 0.02 mg/ml sulfatase from *A. aerogenes* in 50 mM Tris buffer (pH 7.48) at 37 °C. As shown in Table 1 and Additional file 1: Figure S3, the K_m_ and V_max_ values of sulfatase from *A. aerogenes* were determined using probe **1** to be 187 ± 13 μM and 10.9 ± 0.2 μM/min, respectively. To determine the limit of detection (LOD), 1 mM probe **1** was used in 50 mM Tris buffer at 37 °C (pH 7.48). LOD values of probe **1** calculated through fluorescence enhancement were 61 ng/ml for *H. Pomatia* sulfatase and 35 ng/ml for *A. aerogenes* sulfatase (Table 1; Additional file 1: Table S1). On the other hand, the LODs for *H. Pomatia* sulfatase and *A. aerogenes* sulfatase determined by UV absorbance changes at 630 nm after 2 h incubation were 2.5 and 1.99 μg/ml, respectively (Table 1; Additional file 1: Table S1).

**Table 1:** Kinetic parameters of *A. aerogenes* sulfatase and detection limits

	K_m_ (μM)	V_max_ (μM/min)	LOD (FI) (ng/ml)	LOD (UV) (μg/ml)
Probe **1**	187 ± 13	10.9 ± 0.2	35.3 ± 2.6^b^	1.99 ± 0.09^c^
MFS^a^	598 ± 67	(75 ± 4.8) × 10^−6^	158^d^	
RS^a^	162 ± 10	(606 ± 12) × 10^−6^	15.8^d^	
*p*-NPS^a^	1800 ± 120			0.0158^d^

^a^The values were reported in reference, Smith et al. (2014)

^b^The values were calculated by fluorescence intensity changes at 415 nm after 30 min incubation

^c^The values were calculated by UV absorption changes at 630 nm after 2 h incubation

^d^The values were obtained after 10 min incubation

### Colony-based liquid-phase assays

**Fig. 2:**
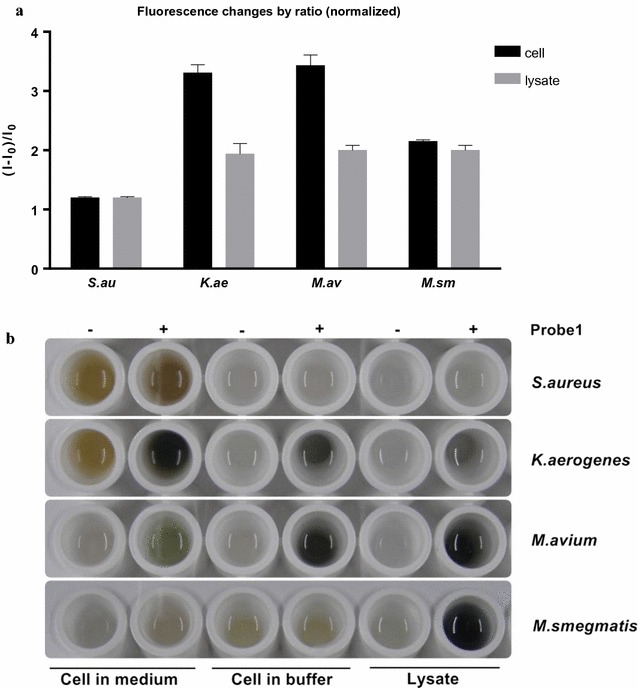
**a** Fluorescence enhancement and **b** color changes of 1 mM probe **1** incubated with *S. aureus, K. aerogenes, M. avium* and *M. smegmatis* colonies and lysates

Four different strains of bacteria were used for the tests. We chose *Klebsiella aerogenes* which is a gram-negative bacteria having periplasmic arylsulfatases, and *M. avium* and *M. smegmatis* possessing arylsulfatase activity which might be related with infection (Mougous et al. 2002). *Staphylococcus aureus* lacking sulfatase genes was used as a control (Sardiello et al. 2005). Colony-based liquid-phase assays were performed by incubating 1 mM probe **1** with bacterial cultures (OD_600_ = 3.0) in 50 mM Tris buffer (Adachi et al. 1974, 1975; Beil et al. 1995; Harada 1964; Okamura et al. 1976). The fluorescence intensities of probe **1** incubated with *K. aerogenes*, *M. avium* and *M. smegmatis* colonies and lysates for about an hour increased by 2- to 3.5-fold (Fig. 2a; Additional file 1: Figure S4), while there were no fluorescence changes of probe **1** incubated with *S. aureus* colonies and lysates that lack sulfatase genes and activity. Moreover, colored precipitates were observed in the lysates of *K. aerogenes*, *M. avium*, and *M. smegmatis* after overnight incubation with probe **1**, while probe **1** incubated with *K. aerogenes* and *M. avium* colonies generated coloured precipitates after 3 days (Fig. 2b). In case of *M. smegmatis* colonies, the fluorescence intensity was slowly increased but colored precipitates were rarely observed (Fig. 2b; Additional file 1: Figure S4). Incubation of probe **1** with *S. aureus* colonies and lysates generated no colored precipitates (Fig. 2). To confirm that the changes in the fluorescence signal was solely due to sulfatases, we measured the specific activity of sulfatases by fluorescence changes of probe **1** incubated with different concentrations of proteins of *M. avium* lysates for 30 min. The values of specific activities at 30 min between different concentrations of the proteins (specific activity of sulfatase = fluorescence intensity/amount of total proteins, see Additional file 1: Figure S4e, f for details. Note that Additional file 1: Figure S4e, f are different sets of experiments) were almost identical, which indicated that the fluorescence enhancement of probe **1** was induced by sulfatase in *M. avium* (Additional file 1: Figure S4).

### Solid-phase colony-based assays

**Fig. 3:**
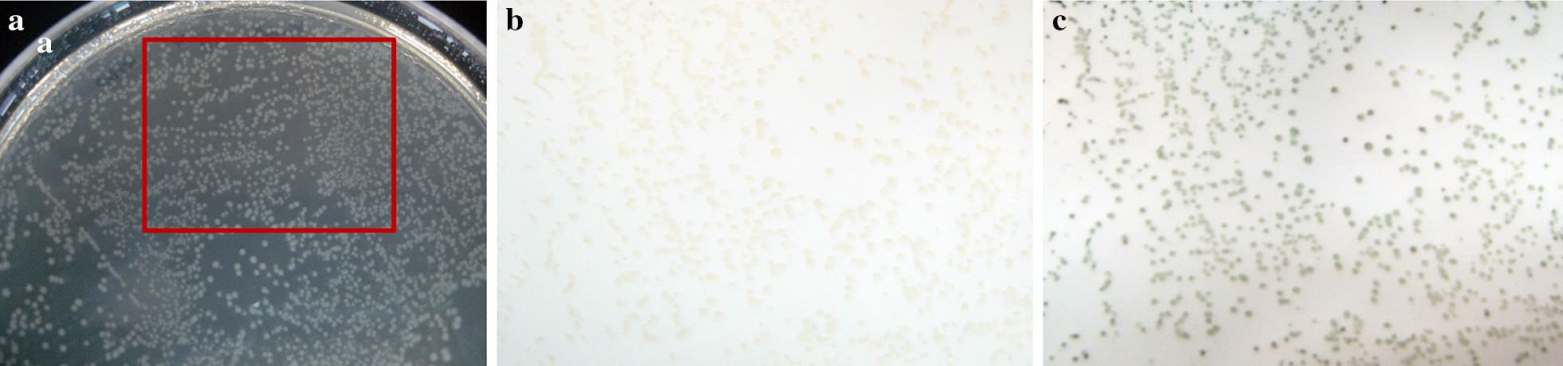
*K. aerogenes* **a** on agar plate, **b** on membrane filters without and **c** with probe **1**

Colonies of *K. aerogenes* on cellulose acetate membrane filters (0.2 μm pore size) were placed on the top of filter papers soaked in 5 mM probe solution and incubated at 37 °C overnight. As shown in Fig. 3, dark colored colonies were observed on the membrane filter of *K. aerogenes* which was placed on the filter papers soaked in probe solution, whereas only faint colonies were visible on the membrane filter of *K. aerogenes* without probe **1**.

## Discussion

We present simple and direct liquid- and solid-phase colony-based assays of bacterial sulfatases using an activity-based probe **1**. Hydrolysis of probe **1** by sulfatases induced fluorescence intensity enhancement and generation of colored precipitates, which enabled monitoring bacterial arylsulfatase activity. The fluorescence enhancement was elicited by the generation of *N*-methyl isoindole upon treatment with the sulfatase (Yoon and Hong 2017), while precipitation might be caused by post-polymerization of *N*-methyl isoindole molecules (Green et al. 2014).

The fact that probe **1** induced the fluorescence enhancement and formation of colored precipitates in a time-dependent manner upon treatment with purified sulfatases (Fig. 1) indicated that probe **1** could be utilized for sulfatase activity assays. As shown in Table 1 and Additional file 1: Table S1, the K_m_ value obtained using probe **1** was comparable to those obtained using previously reported fluorogenic sulfatase activity probes, 3-*O*-methylfluorescein-sulfate (MFS) and resorufine-sulfate (RS) (Smith et al. 2014). Comparing to the V_max_ values obtained using MFS and RS which were determined to be 1.25 ± 0.08 pmol/s and 10.1 ± 0.2 pmol/s (Smith et al. 2014), probe **1** reacted with sulfatase from *A. aerogenes* more rapidly. In addition, considering the incubation time, the LOD values measured by probe **1** would be similar to the reported values by fluorogenic probes, MFS (158 ng) and RS (15.8 ng), but the LOD value measured by UV absorbance of probe **1** was larger than that by a chromogenic probe, *p*-NPS (15.8 ng) (Smith et al. 2014). This is presumably due to the post-polymerization of *N*-methyl isoindole, which was generated and accumulated through the hydrolysis of probe **1** by sulfatases. In spite of the less sensitivity and longer incubation time for the observation of color changes, probe **1** could also provide an easy and simple detection method through naked eye. Therefore, probe **1** is a good substrate for bacterial sulfatases and could detect sulfatase activity by fluorometric and colorimetric assays.

We applied probe **1** to microorganisms to demonstrate the feasibility to detect arylsulfatase activity in colony-based liquid-phase assays. Fluorescence enhancement of probe **1** with *K. aerogenes*, *M. avium* and *M. smegmatis* (Fig. 2a; Additional file 1: Figure S4) indicated that the bacterial colonies and lysates having sulfatase activity hydrolyzed the sulfate ester of probe **1** to generate fluorescent *N*-methyl isoindole. Colored precipitates were also observed except in the well of probe **1** with *M. smegmatis* colonies (Fig. 2b). This is presumably because the sulfate ester moiety of probe **1** was so slowly hydrolyzed by *M. smegmatis* colonies that *N*-methyl isoindole insufficiently generated could not be effectively polymerized (Additional file 1: Figure S4). As a result, it was implied that the sulfate ester of probe **1** was cleaved to generate a fluorescent *N*-methyl isoindole by colonies and lysates of *K. aerogenes*, *M. avium* and *M. smegmatis* having sulfatase activity, and colored precipitates were formed by a sufficient amount of *N*-methyl isoindole. In contrast, probe **1** did not react with *S. aureus* which lacks sulfatase genes. Therefore, probe **1** can be used to detect sulfatase activity of bacteria through fluorescence enhancement and naked eye in liquid-phase colony-based assays.

Finally, we tested the accessibility of probe **1** for colony-based solid-phase assays. Solid-phase assays allowed accurate staining, thus enabling the identification of target locations and the selection of target colonies under heterogeneous conditions. The results shown in Fig. 3 indicate that probe **1** is suitable for colony-based solid-phase assays as its treatment with sulfatase leads to the formation of an insoluble colored product.

In summary, an activity-based probe **1** was successfully used for detection of bacterial arylsulfatase activity in liquid- and solid-phase assays. Probe **1** was hydrolyzed by bacterial sulfatase, generating *N*-methyl isoindole that leads to fluorescence enhancement and colored precipitates. Although longer incubation time and enough accumulation of *N*-methyl isoindole were required to generate colored precipitates, probe **1** is applicable to bacterial sulfatase activity assays in liquid- and solid-phase through the observation of color changes as well as fluorescence enhancements. Consequently, our probe may offer a simple method for screening and sorting of the potential bacterial colonies having arylsulfatases activity.

## Additional file

**Additional file 1.** Supplementary information.

## Abbreviations

BSA: bovine serum albumin
OD: optical density
DMSO: dimethyl sulfoxide
LOD: limit of detection
