# Supplementary material for: Detection of bacterial sulfatase activity through liquid- and solid-phase colony-based assays
Source: AMB Express. 2017 Jul 11;7:150. doi: 10.1186/s13568-017-0449-3 (PMC5503846; doi:10.1186/s13568-017-0449-3)
Supplement: Supplementary file 1 — Additional file 1. Supplementary information. [file 13568_2017_449_MOESM1_ESM.pdf]

Supplementary Information for:

Detection of bacterial sulfatase activity through liquid- and solid-phase colony-based assays

Hey Young Yoon[a,1], Hyung Jun Kim[b, 1], Soojin Jang[b] and Jong-In Hong[a,*]

[a]*Department of Chemistry, College of Natural Sciences, Seoul National University, Seoul 151-747, Republic of Korea*

[b]*Department of Discovery Biology, Antibacterial Resistance Research Laboratory, Institut Pasteur Korea, 16 Daewangpangyo-ro 712 beon-gi, Bundang-gu, Seongnam-si, Gyeonggi-do, 13488, Republic of Korea*

Corresponding authors:

Prof. Jong-In Hong

E-mail: jihong@snu.ac.kr

Tel: +82 874 5902; Fax: +82 2 889 1568

Hey Young Yoon: yhy7748@snu.ac.kr

Hyung Jun Kim: hyungjun.kim@ip-korea.org

Soojin Jang: soojin.jang@ip-korea.org

[1] These authors contributed equally.

Contents

General procedures...S2

Preparations ……………………………………………………………………..…………...S2

Optical responses by purified sulfatases …..………...S5

Enzyme Kinetics ………………………………………………………..................................S6

References………………………………………………………………………….……………S8

1. General

^{1}H and ^{13}C NMR spectra were taken on a Bruker Advance DPX-300 or a Bruker Advance 500 spectrometer. Chemical shifts are given in parts per million using as internal reference the residual resonances of deuterated solvents (^{1}H NMR chemical shifts: δ = 7.27 ppm for $CDCl_3$, δ = 2.05 ppm for acetone-d_6 and δ = 2.50 ppm for $(CD_3)_2SO$) (^{13}C NMR chemical shifts: δ = 77.23 ppm for $CDCl_3$, δ = 205.87, 30.60 ppm for acetone-d_6 and δ = 39.51 ppm for $(CD_3)_2SO$). Fast atom bombardment mass spectrometry (FAB-MS) data were obtained using a JEOL JMS-AX505WA mass spectrometer with m-nitrobenzyl alcohol (NBA) as a matrix and were reported in units of mass to charge (m/z). Analytical thin layer chromatography was performed using Kieselgel 60F-254 plates from Merck. Column chromatography was carried out on Merck silica gel 60 (70-230 mesh). UV/vis spectra were collected on a Beckman DU-800 and fluorescent spectra were collected on a Jasco FP-6500 and SpectraMax M2 spectrophotometer at 37°C unless noted otherwise. Ultrasonications were carried out on Fisher 550 Sonic Dismembrator and centrifugation was carried out on Combi-514 of Hanil Sciences Industrial Cooperation.

All chemical reagents and sulfatases from *Helix pomatia,* (S9626 Sigma) and *Aerobacter aerogenes* (S1629 Sigma) were purchased from either Sigma-Aldrich or TCI and used without any further purification.

2. Preparations

2-1. Chemical synthesis

We synthesized probe **1** as follows (Yoon and Hong 2017):

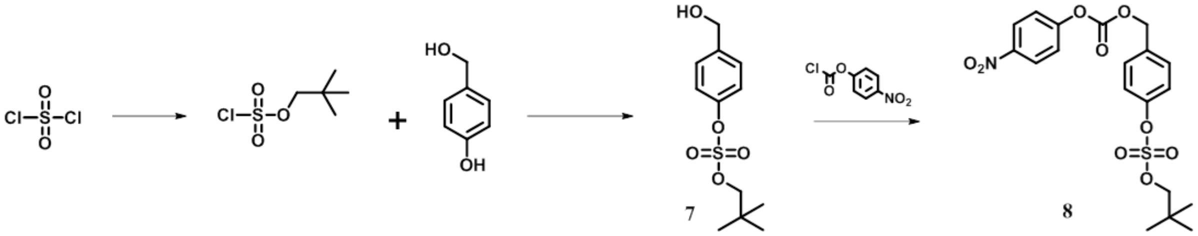

A solution of sulfuryl chloride (1.62 ml, 20 mmol) in Et_2O was cooled to -75°C under nitrogen. A solution of neopentyl alcohol (1.76 g, 1 equiv.) and pyridine (1.62 ml, 1 equiv.) in Et_2O was added dropwise to the cooled solution for 1 h. The resulting mixture was warmed to room temperature and stirred for an additional 2 h. The resulting white precipitates were filtered off and the filtrate was concentrated in vacuo. The product was used for a next synthesis step without further purification.

Compound 7: To a solution of 4-hydroxybenzylalcohol (1.7 g, 1 equiv.) in anhydrous THF, sodium hydride (437 mg, 1.1 equiv.) was slowly added at 0°C. After 10 min, the crude neopentyl chlorosulfate was added to the reaction mixture. Then, the reaction mixture was warmed to room temperature, and stirred overnight. Upon completion, sodium hydride in the reaction mixture was quenched with water and THF was removed in vacuo. Ethyl acetate was added to the residue and the organic layer was washed with brine, then dried over Na_2SO_4 and concentrated in vacuo. The crude product was purified by silica gel chromatography (chloroform:acetone = 50:1) to give compound **7** (970 mg, 17% yield). ^{1}H NMR (300 MHz, $CDCl_3$) δ 1.02 (9H, s), 4.10 (2H, s), 4.74 (2H, s), 7.31 (2H, d, J=8.7Hz), 7.44 (2H, d, J = 8.5Hz).

Compound 8: To a solution of 4-nitrophenyl chloroformate (640 mg, 1.1 equiv.) in anhydrous THF was added pyridine (257 µl, 1 equiv.) at 0°C. After stirring for 20 min, a solution of compound **7** (970 mg, 3.54 mmol) in

anhydrous THF was added dropwise within 10 min, and the mixture was allowed to warm up to room temperature. Stirring was continued at room temperature for 16 h and then, the solvent was removed in vacuo, the residue were dissolved in ethyl acetate. The resulting solution was washed with saturated NH_4Cl aqueous solution several times and concentrated in vacuo. The crude product was purified by silica gel column chromatography (chloroform:acetone = 100:1) to give compound **8** (784 mg, 1.78 mmol, 50% yield). ^{1}H NMR (300 MHz, $CDCl_3$) δ 1.03 (9H, s), 4.13 (2H, s), 5.32 (2H, s), 7.38 (2H, d, *J* = 8.5 Hz), 7.41 (2H, d, *J* = 9.0 Hz), 7.52 (2H, d, *J* = 8.6 Hz), 8.30 (2H, d, *J* = 8.5 Hz); ^{13}C NMR (300 MHz, $CDCl_3$) δ 25.86, 31.90, 69.75, 83.69, 115.331, 121.43, 121.81, 125.28, 125.46, 130.30, 133.64, 145.39, 150.48, 155.45; HRMS (FAB): m/z calcd. for $[C_{19}H_{21}NO_9S+Na^+]$ 462.0835, found 462.0838.

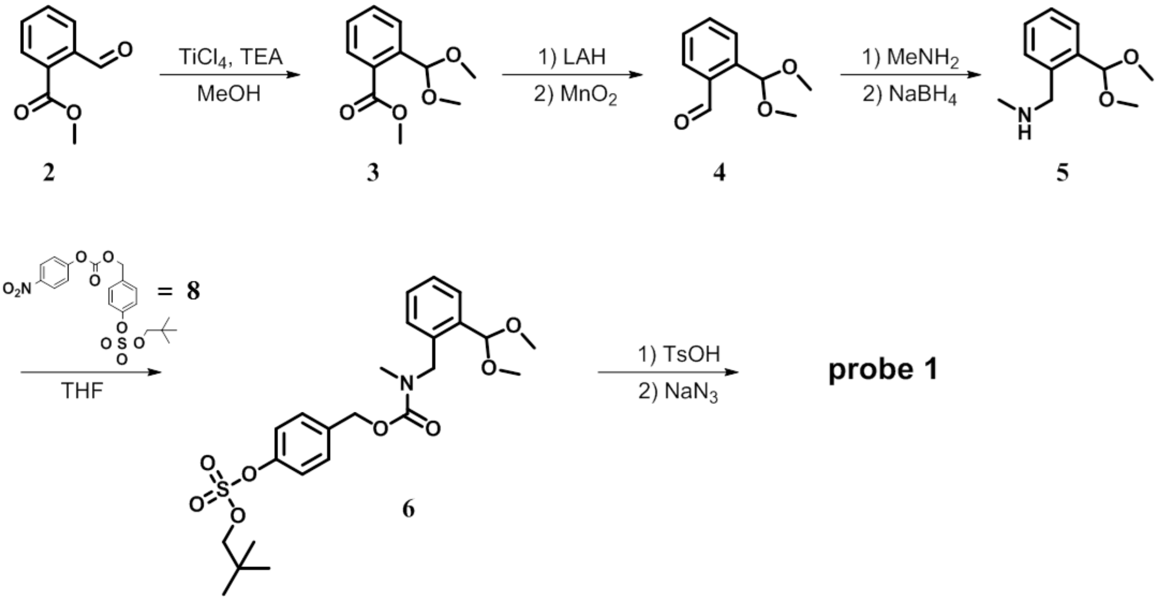

Compound 3: To a solution of methyl 2-formylbenzoate (**2**) (1.62 g, 9.87 mmol) in methanol (20 ml) at 0°C under N_2 was added 1 M $TiCl_4$ (1 ml, 0.1 eqiuv.) solution in CH_2Cl_2 in one portion. After being stirred for 30 min, the resulting mixture was treated with triethylamine (3 ml, 10 equiv.), and was stirred for an additional 3 h at room temperature. After methanol was removed in vacuo, the residue was dissolved in ethyl acetate and washed with brine 3 times, dried over Na_2SO_4 and evaporated. The crude residue was purified through silica gel column chromatography (hexane:EA = 5:1) to give compound **3** (1.8 g, 8.56 mmol, 87% yield). ^{1}H NMR (300 MHz, $CDCl_3$) δ 3.39 (6H, s), 3.93 (3H, s), 6.08 (1H, m), 7.40 (1H, t, *J* = 7.5 Hz), 7.53 (1H, t, *J* = 7.6 Hz), 7.76 (1H, d, *J* = 7.7 Hz), 7.82 (1H, d, *J* = 7.6 Hz).

Compound 4: To a suspension of lithium aluminum hydride (250 mg, 2 equiv.) in anhydrous THF was added **3** (900 mg, 4.3 mmol) dissolved in anhydrous THF at 0°C slowly under N_2. The reaction mixture was stirred at room temperature for 5 h. Then, the solution was cooled down to 0°C and quenched by 1 M aqueous NaOH solution. The mixture was dried with Na_2SO_4 and filtered through a pad of Celite. The filtrate was concentrated in vacuo. The reduction product was used for the next synthetic step without further purification.

To a solution of the reduction product (653 mg, 3.59 mmol) dissolved in CH_2Cl_2 was added MnO_2 (3.7 g, 10 equiv.). After being stirred at room temperature overnight, the mixture was filtered with Celite and silica pad. The filtrate was concentrated in vacuo and the residue was purified with silica column chromatography (hexane:EA = 3:1) to give **4** (540 mg, 2.98 mmol, 69% yield). ^{1}H NMR (300 MHz, $CDCl_3$) δ 3.42 (6H, s), 5.90 (1H, s), 7.52 (1H, t, *J* = 7.3 Hz), 7.62 (1H, t, *J* = 7.4 Hz), 7.70 (1H, d, *J* = 7.5 Hz), 7.95 (1H, d, *J* = 7.5 Hz), 10.46 (1H, s).

Compound 6: To a solution of **4** (540 mg, 2.98 mmol) in MeOH was added 2M solution of methylamine in THF (4 ml, 2 equiv.). The resulting mixture was stirred at room temperature overnight, cooled down to 0℃, and treated with sodium borohydride (570 mg, 5 equiv). The mixture was stirred for 1 h and then, quenched by water. After methanol was removed in vacuo, the residue was dissolved in ethyl acetate and washed with aqueous $NaHCO_3$ and brine 3 times, dried over Na_2SO_4 and concentrated in vacuo. Product **5** was used for the next synthetic step without further purification (219 mg).

To a solution of **5** (89 mg, 0.46 mmol) in THF were added TEA (192 µl, 3 equiv.) and **8** (200 mg, 1equiv.). The mixture was stirred at room temperature overnight and concentrated in vacuo, and residue was dissolved in ethyl acetate and washed with aqueous $NaHCO_3$ and saturated NH_4Cl solution, dried over Na_2SO_4 and concentrated. The crude residue was purified by silica column chromatography (chloroform:acetone = 50:1) to give **6** (107 mg, 0.22 mmol, 47% yield). ^1H NMR (300 MHz, $CDCl_3$) δ 1.02 (9H, s), 2.92 (3H, d, *J* = 24 Hz), 3.31(6H, d, *J* = 10 Hz), 4.10 (2H, s), 4.69 (2H, s), 5.19 (2H, d, *J* = 15.6 Hz) 5.41 (1H, d, *J* = 23Hz), 7.16-7.23 (2H, br), 7.31 (3H, m), 7.46 (1H, m), 7.55 (1H, s).

Probe 1: To solution of **6** (57 mg, 0.12 mmol) in acetone was added *p*-toluenesulfonic acid monohydrate (6.5 mg, 0.3 equiv.) and stirred at room temperature for 2 h. Acetone was removed in vacuo and the residue was purified through silica column chromatography (chloroform:acetone = 30:1). Then, the resulting product (50 mg, 0.11 mmol) and sodium azide (10 mg, 1.2 equiv.) were dissolved in DMF. The solution was stirred and heated at 70℃ overnight. Solvent was removed in vacuo and the crude product was purified by silica gel column chromatography (CH_2Cl_2:methanol = 10:1) gave probe **1** as the sodium salt (37 mg, 0.098 mmol, 88% yield). ^1H NMR (300 MHz, CD_3OD) δ 2.95 (3H, s), 4.96 (2H, s), 5.12 (2H, d, *J* = 21 Hz) 7.21-7.39 (5H, m), 7.52 (1H, t, J = 7.3 Hz), 7.60 (1H, s), 7.92 (1H, d, *J* = 7.1 Hz), 10.15 (1H, d, *J* = 19 Hz); ^{13}C NMR (75 MHz, $CDCl_3$) δ 29.38, 49.67, 66.68, 121.11, 125.85, 127.45, 128.74, 133.58, 133.58, 133.91, 152.35, 193.72; HRMS (FAB): m/z calcd. for [$C_{17}H_{16}NNaO_7S$ + H^+] 402.0623, found 402.0621.

2-2. Lysis

The fully-grown bacterial culture was centrifuged at 3300 rpm for 10 minutes. The collected pellets were washed three times with distilled water and then re-suspended in 50 mM Tris-buffer (pH 7.4) with a final OD_{600} of 3.0. To obtain lysate of the bacteria, 300 µl of the re-suspension was added to a 1.5 ml sonication tube and then sonicated for 5 minutes. After the sonication, centrifuge was applied and the supernatant was transferred to a new tube. The Bradford method was used to determine the protein concentration of the lysate.

3. Experiments

3-1. Optical response of probe 1 by purified sulfatases

Biochemical activity assays with *H. Pomatia* sulfatase (Sigma, S9226) and *A. aerogenes* sulfatase (Sigma, S1629) were carried out in 96-well plates with the total volume of each plate being 250 μl at 37°C and pH 7.4. Stock solution of *H. pomatia* sulfatases was prepared to be 0.625 mg/ml in 50 mM Tris buffer (500 mM NaCl, 1 mM $MgCl_2$, 1 mM $CaCl_2$, pH 7.4) and stock solution of probe **1** was prepared to be 100 mM in DMSO. Then, 1 mM probe and various amounts of sulfatase in 50 mM Tris buffer were used. Fluorescence intensity (λ_{ex}=327 nm, λ_{em}=415 nm) was measured in a time-dependent manner (0 – 165 min.).

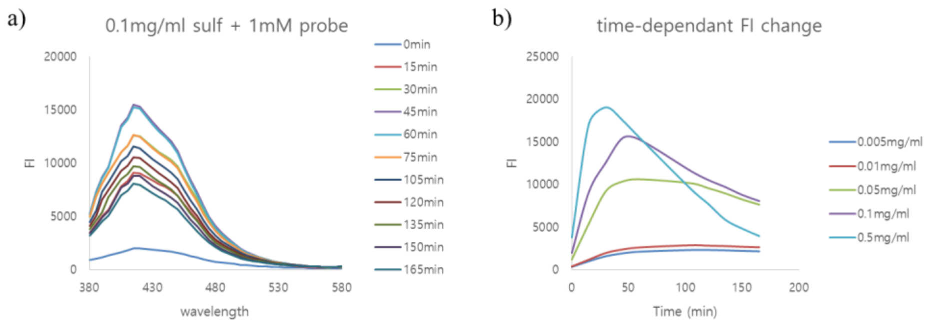

Figure S1. (a) Fluorescence changes of 1 mM probe **1** with 0.1 mg/ml of sulfatase, (b) time-dependent fluorescence changes of 1 mM probe **1** with various amounts of sulfatase.

For the UV absorbance studies, 1 mM probe **1** with various amounts of sulfatase was incubated at 37°C. Stock solution of *A. aerogenes* sulfatase was prepared to be 0.05 mg/ml in 50 mM Tris buffer (500 mM NaCl, 1 mM $MgCl_2$, 1 mM $CaCl_2$, pH 7.4).

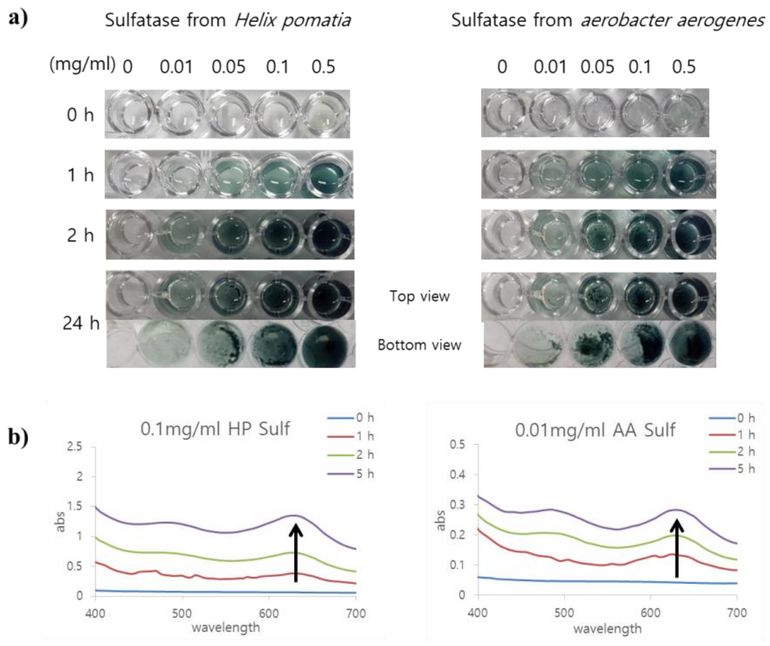

Figure S2. (a) Formation of colored precipitates upon incubation of 1 mM probe **1** with various concentrations of sulfatase from *H. pomatia* (left) and *A. aerogenes* (right) at 37°C for 1 h, 2 h and 24 h, b) time-course UV absorption changes upon treatment of probe **1** with sulfatase from *H. pomatia* (left) and *A. aerogenes* (right).

3-2. Enzyme kinetics and limits of detection

Kinetic experiments of probe **1** with *A. aerogenes* (AA) sulfatase were carried out at 37 ℃ in 50 mM Tris buffer (pH 7.48). The fluorescence intensities of a series of different concentrations of probe **1** (0, 10, 20, 50, 100, 200, 500, 1000 and 2000 μM) with AA sulfatase (0.02 mg/mL) in a 96-well black bottom plate were measured in a time-dependent manner. The rate of increase in fluorescence intensity at 415 nm was used to determine the kinetic parameters of enzyme hydrolysis. The kinetic parameters (K_M and V_{max}) were determined from triple-reciprocal plots of hydrolysis rates versus substrate concentration (Michaelis-Menten plot).

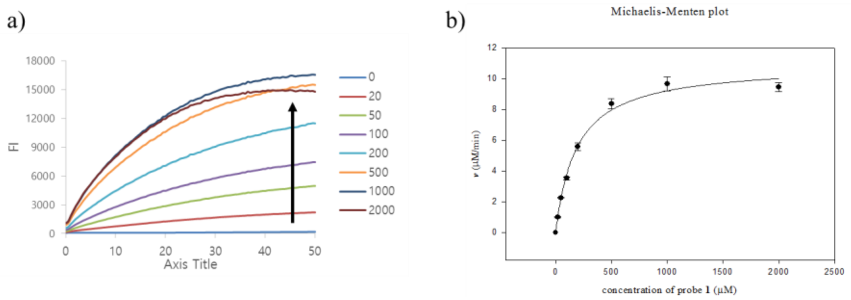

Figure S3. a) Fluorescence intensity enhancement of probe **1** in various concentrations incubated with AA sulfatase in a time-dependent manner, b) Michaelis-Menten plot.

Table S1. Kinetic parameters of *H. pomatia* sulfatase and LOD of probe **1**

	K_m (μM)	V_{max} (μM/min)	LOD [b] (ng/ml)	LOD [c] (μg/ml)
H. pomatia	203± 57[a]	0.27 ± 0.06 [a]	61.4 ± 0.2	2.50 ± 0.02

[a] The values were reported in reference (Yoon and Hong 2017).

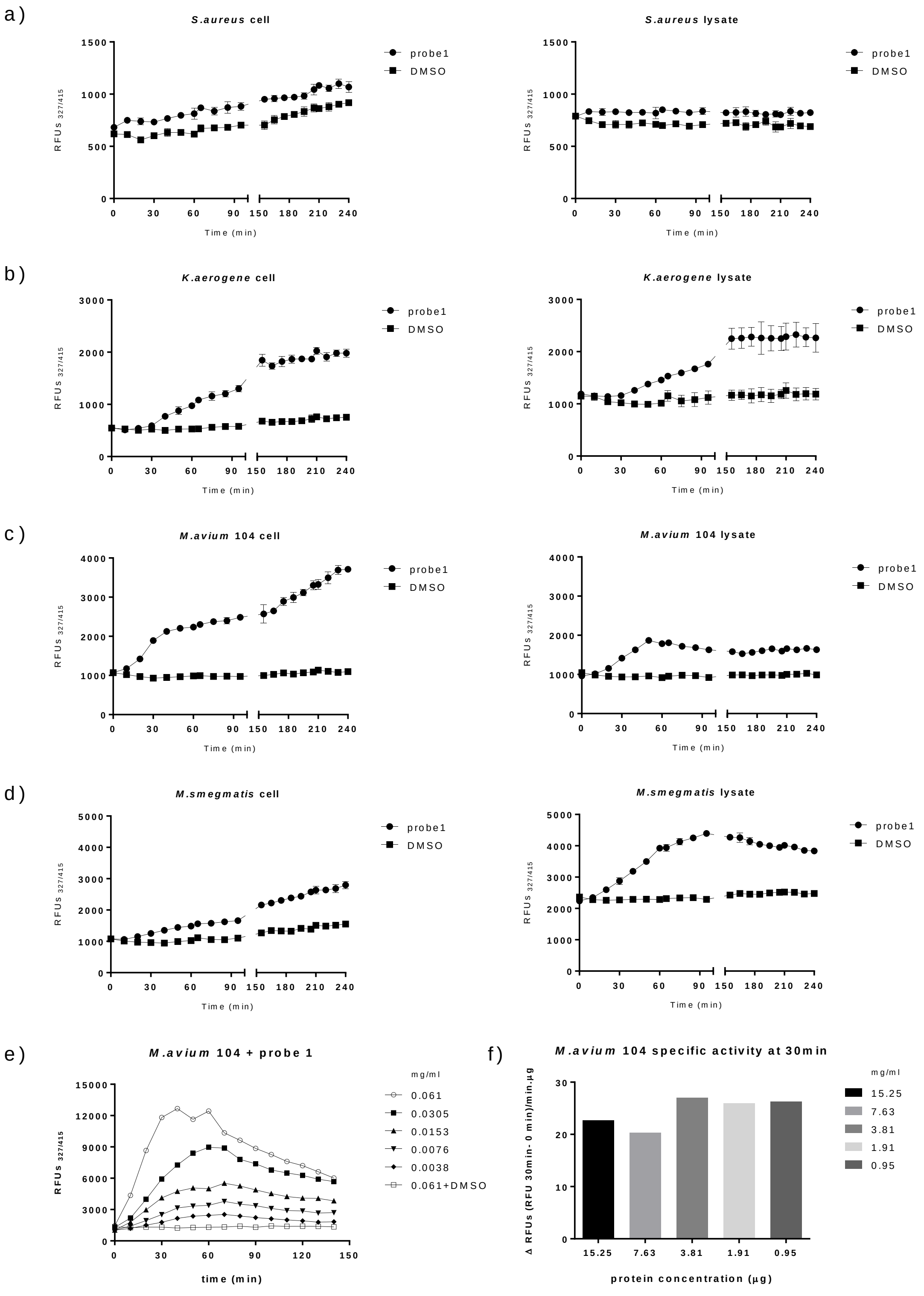

Figure S4. Fluorescence intensity enhancement of probe **1** incubated with a) *S. aureus* colony (left) and lysate (right, 4.2 μg of protein/ml), b) *K. aerogenes* colony (left) and lysate (right, 40.3 μg of protein/ml), c) *M. avium* colony (left) and lysate (right, 4.5 μg of protein/ml), d) *M. smegmatis* colony (left) and lysate (right, 19.5 μg of protein/ml), e) fluorescence enhancement of probe **1** upon treatment of different protein concentrations of *M. avium* lysate, and f) specific activity of sulfatase in *M. avium* measured by probe **1**.

Reference

Yoon HY, Hong JI (2017) Sulfatase activity assay using an activity-based probe by generation of *N*-methyl isoindole under reducing conditions. Anal Biochem 526:33-38 doi:10.1016/j.ab.2017.03.012
